# An observational cohort study of the use of five-grass-pollen extract sublingual immunotherapy during the 2015 pollen season in France

**DOI:** 10.1186/s13223-018-0262-9

**Published:** 2018-09-24

**Authors:** Patrick Blin, Pascal Demoly, Martine Drouet, Bruno Falissard, Séverine Lignot-Maleyran, Hélène Maizi, Simon Lorrain, Régis Lassalle, Cécile Droz-Perroteau, Nicholas Moore, Mathieu Molimard

**Affiliations:** 1 Bordeaux PharmacoEpi, Université de Bordeaux, 146, rue Léo Saignat, 33076 Bordeaux Cedex, France; 2 CIC Bordeaux, CIC Bordeaux CIC1401, Bordeaux Cedex, France; 3 Allergy Department, CHRU de Montpellier, Montpellier, France; 4 Pneumonology Department, CHU d’Angers, Angers, France; 5 CESP/INSERM U1018, Maison de Solène, Paris, France; 6 CHU INSERM U1219, Bordeaux, France; 7 Pharmacology Department, CHU de Bordeaux, Bordeaux, France

**Keywords:** Allergic rhinitis, Sublingual immunotherapy, Pollen season, France

## Abstract

**Background:**

Allergic rhinitis affects around one quarter of the Western European population. Prophylactic allergen immunotherapy may be useful to reduce the risk of acute symptomatic attacks (hayfever). A five-grass pollen extract sublingual immunotherapy (5GPE-SLIT) has been developed for the treatment of allergic rhinitis to grass pollen. The objective of this study was to describe real-world treatment patterns with 5GPE-SLIT in France with respect to the prescribing information.

**Methods:**

This prospective cohort study was conducted by 90 community and hospital allergists. Adults and children (> 5 years old) starting a first treatment with 5GPE-SLIT prior to the 2015 pollen season were eligible. Data was collected at the inclusion visit and at the end of the pollen season. The primary outcome variable was compatibility of 5GPE-SLIT prescription with the prescribing information. This was determined with respect to four variables: (1) interval between 5GPE-SLIT initiation and onset of the pollen season ≥ 3 months, (2) age of patient ≥ 5 years, (3) intermittent symptoms or mild symptom severity (4) confirmatory diagnostic test. At study end, symptoms reported during the pollen season and any modifications to treatment or adverse events were documented.

**Results:**

280 adults and 203 children were enrolled. The prescribing information was respected for 82.5% of adults and 86.7% of children. A skin test was performed for all patients. 5GPE-SLIT was started 3–5 months before the pollen season for 85.3%. Treatment was discontinued before the start of the pollen season in 11.0% of patients overall, generally because of an adverse event (78.8% of discontinuations). The mean duration of treatment was 5.2 months in adults and 5.6 months in children. At the end of follow-up, symptoms during the pollen season were intermittent for 75.0% of adults and 85.7% of children, and severity was mild for 61.8 and 66.0% respectively. During 5GPE-SLIT, the following symptoms reported during the previous year were not reported again in > 50% of patients: nasal congestion, rhinorrhoea, repeated sneezing, conjunctivitis and nasal pruritus.

**Conclusions:**

5GPE-SLIT use was generally consistent with prescribing recommendations and was associated with an improvement of AR severity, with resolution of the principal AR symptoms in around half the patients treated.

*Trial registration* EUPAS9358. Registered 13 May 2015. Not prospectively registered. http://www.encepp.eu/encepp/viewResource.htm?id=16229

**Electronic supplementary material:**

The online version of this article (10.1186/s13223-018-0262-9) contains supplementary material, which is available to authorized users.

## Background

Allergic rhinitis is a frequent and debilitating condition characterised by inflammation of the nasal epithelium in response to an airborne environmental allergen, leading to symptoms of rhinorrhoea, repetitive sneezing, blocked nose and nasal irritation [1, 2]. The prevalence of clinically-confirmed allergic rhinitis in the general population in Western Europe has been estimated as 23% (24.5% in France) [3], and other general population studies in France using screening questionnaires in telephone interviews have yielded similar prevalence rates [4]. Allergic rhinitis is associated with deteriorated quality of life [5–7] and comorbidity with a number of other conditions, notably asthma and sleep disturbances [7, 8]. The allergic rhinitis and its impact on asthma (ARIA) working group has proposed a classification of allergic rhinitis into persistent versus intermittent and mild versus moderate-severe, which is now generally accepted [2]. In Western Europe, grass pollens are a major source of allergens responsible for allergic rhinitis, [9] with around half of all subjects with allergic rhinitis identified in a general population survey testing positive for grass-pollen-specific IgE [3]. A survey in South-East France found that pollen-specific IgEs were detectable in 11% of a random sample of the general population [10].

Historically, conventional treatment of allergic rhinitis has been principally symptomatic, through use of oral or topical antihistamines as needed to relieve symptoms [11]. In patients with moderate or severe persistent allergic rhinitis, the most recent ARIA guidelines recommend long-term prophylactic therapy with intranasal corticosteroids, a second-generation antihistamine or a leukotriene antagonist [2]. In case of failure of pharmacotherapy, allergen immunotherapy should be considered [2]. Allergen immunotherapy involves administering high doses (either directly of after up-titration from on initial low dose, of a specific allergen source in order to induce tolerance to it by rebalancing the immune system and thereby to reduce the risk of a symptomatic allergic reaction when the patient is exposed to the causative agent [11, 12]. Allergen immunotherapy can be administered by either the subcutaneous (SCIT) or sublingual (SLIT) route. The sublingual route has been better validated in clinical trials and appears to be safer [13]. A number of SLIT preparations have been introduced into clinical practice over the last decade.

In the case of allergic rhinitis triggered by grass pollen, a SLIT preparation has been developed containing pollen allergens from five common grasses, namely cock’s-foot (*Dactylis glomerata* L.), sweet vernal grass (*Anthoxanthum odoratum* L.), perennial ryegrass (*Lolium perenne* L.), common meadow-grass (*Poa pratensis* L.) and meadow cat’s-tail (*Phleum pratense* L.). This five-grass pollen extract SLIT (5GPE-SLIT) is administered in the form of 100 and 300 mg sublingual tablets around 4 months before the beginning of the pollen season until the end of the season.

The efficacy and safety of 5GPE-SLIT has been demonstrated in three large randomised clinical trials [14–16] to be effective in preventing symptoms of allergic rhinitis due to grass pollen and to be well tolerated. In consequence, this treatment is now approved in Europe and in the USA as a prescription medication for the treatment of grass pollen allergic rhinitis with or without conjunctivitis in adults, adolescents and children (above the age of 5) with clinically relevant symptoms, confirmed by a positive skin test or a positive titre of specific grass pollen IgE.

However, there is little information on how 5GPE-SLIT is used in everyday clinical practice or on its effectiveness and tolerability outside the context of a clinical trial. The primary objective of the present study was to describe indications for 5GPE-SLIT, treatment period and concomitant allergy medications under real-world conditions during the 2015 pollen season. Secondary objectives were to describe the characteristics of the prescribing physicians and the treated patients and to document treatment discontinuation and the occurrence of any adverse events.

## Methods

This was a prospective cohort study conducted in France of patients starting treatment with 5GPE-SLIT for the 2015 pollen season and followed throughout the pollen season. The conduct of the study was supervised by a steering committee composed of two allergists and a biostatistician. The study was mandated by the French Health Technology Assessment agency (HAS; *Haute Autorité de la Santé*) as a post-marketing commitment at the time of approval of the medication in France, and the study protocol was approved by the HAS.

### Participating physicians

The study was conducted by community and hospital allergists identified from an exhaustive national list. All allergists on this list were contacted and invited to participate in the study and all allergists who accepted to participate were enlisted.

### Patients

Each participating physician was expected to enrol all patients fulfilling the eligibility criteria attending a consultation during a 3 month period between December 2014 and February 2015 (prior to the 2015 pollen season), up to a maximum of ten patients per physician. Adults and children (aged 5–18 years) who were starting a first treatment with 5GPE-SLIT and who agreed to participate in the study were eligible. Patients previously treated by 5GPE-SLIT, participants in clinical trials, patients unable to complete the study questionnaires and patients who were not expected to be followed throughout the pollen season (for example, patients planning to move house) were excluded.

A reference set of patients representative of all those prescribed 5GPE-SLIT in France was obtained using a health insurance database, the *Echantillon Généraliste de Bénéficiaires* (EGB), which represents a permanent sample of individuals selected at random from 1/97th of the nationwide French claims database, consisting of around 700,000 individuals representative of the French population. The EGB database contains information on all eligible medical expenditure reimbursed for each insurance beneficiary, including all medication prescribed and the date of prescription. All patients in the EGB database prescribed 5GPE-SLIT for the first time during the study period (December 2014 to February 2015) were identified and information extracted on their demographic characteristics and on all prescription of allergy medications.

### Study procedures and data collection and outcome variables

Since this was an observational study, there were no protocol-specified study procedures. Data was collected at the inclusion visit and at the end of the study (end of the pollen season). For patients who did not return for a follow-up consultation at the end of the pollen season, they were contacted by telephone by the study coordinating centre whenever possible.

Data was collected on paper questionnaires by the physician and by the patient. The physician completed a medical questionnaire at the inclusion visit and at the end of study visit. The patient completed an auto-questionnaire at the inclusion visit and another at the end of study visit.

Variables collected on the physician medical questionnaire included sociodemographic data on the patient, allergy history, comorbidities, previous allergy treatments, date of initiation of 5GPE-SLIT and dose, concomitant medications and, for the end-of-study questionnaire, any modifications to treatment or adverse events reported during the pollen season. Whether a diagnostic test had been performed was documented together with the class of test used (skin prick test, specific IgE assay, nasal irritation test) and whether the test was positive or not. Participating physicians followed their routine practice with respect to the exact nature of the test (e.g. type of pollen used) and the criterion for positivity (e.g. weal size). The physician also provided details of their own allergology qualifications, where they practiced and their age and gender. At inclusion, the patient rated the personal importance of 25 treatment needs on a five-point Likert scale (Patient Needs Questionnaire; PNQ) and then, at the end of the study, rated the extent to which these same needs were met by treatment (Patient Benefit Questionnaire; PBQ). The PNQ-PBQ has previously been validated in patients with allergic rhinitis [17] and was used here in a validated French translation. A treatment impact score, which could range from 0 (no impact) to 4 (major benefit), was generated for each patient using the data from the PNQ-PBQ [17, 18].

### Outcome variables

The primary outcome variable was the compatibility of prescription with the recommendations in the prescribing information. These recommendations specify the indication for 5GPE-SLIT as “*Treatment of grass pollen allergic rhinitis with or without conjunctivitis in adults, adolescents and children (above the age of 5) with clinically relevant symptoms, confirmed by a positive cutaneous test and/or a positive titre of the specific IgE to the grass pollen*.” Compatibility was determined on the basis of four criteria, namely (1) an interval between 5GPE-SLIT initiation and the onset of the pollen season ≥ 3 months, (2) age of patient ≥ 5 years, (3) symptom frequency rated as intermittent or symptom severity rated as mild (4) diagnosis confirmed by a positive skin-prick test and/or a positive specific IgE assay. The time of initiation of 5GPE-SLIT with respect to the onset of the pollen season was calculated by comparing the date of the inclusion visit at which 5GPE-SLIT was prescribed and the beginning of the pollen season as declared by the French aerobiology surveillance network (*Réseau National de Surveillance Aérobiologique*; RNSA). Allergic rhinitis symptoms were categorised as intermittent or persistent and mild or moderate-severe based on the 2001 ARIA classification [19]. Medications used were classified according to the ATC system [20] and divided into maintenance treatments, taken every day to prevent occurrence of allergic symptoms and acute treatments taken as needed to control allergy symptoms. Adverse events occurring during the 2015 pollen season were documented at the final study visit (or at any unprogrammed intermediate visit) and were classified according to the terminology of the Medical Dictionary for Regulatory Activities (MedRA; 2016 version).

### Statistics

The target population was established at 450 patients (300 adults and 150 children) in order to determine the principal study variables with a precision of 2.5–5.7% for observed frequencies of 5–50%. In order to achieve this target, it was anticipated that ninety participating physicians would need to recruit an average of five patients each. Assuming that certain physicians who accepted to participate would not in fact recruit any patients, the target number of participating physicians was 120 (75% of active participants). The analysis was performed on the analysable population, defined as all patients fulfilling the eligibility criteria, for whom data were available at the inclusion visit and at the end of study visit. Separate analyses were performed for adults aged ≥ 18 years and children. A comparison between the analysable patient set and all patients prescribed 5GPE-SLIT in the EGB database was performed. The analysis was purely descriptive. Continuous variables are described as mean values with standard deviation (SD), median and range. Categorical and ordinal variables are presented as frequency counts and percentages. 95% confidence intervals (95% CI) were calculated for the principal variables of interest. Missing data were not imputed. All analyses were performed using SAS^®^ software version 9.3.

### Ethical considerations

The study protocol received all legal authorisations from the National Medical Council (CNOM), the National Advisory Committee on Medical Research Information (CCTIRS) and the French national data protection agency (CNIL). Since the conduct of the study had no influence on patient care and there were no specific study interventions, ethical committee clearance was not required. All patients provided written informed consent to be contacted by the study coordinating centre if necessary, and all nominative data collected in the study questionnaires was rendered anonymous before entry into the study database. The study was registered with the EUPAS registry as 9358 and conducted according to ENCePP guidance on methodological standards in pharmacoepidemiology.

## Results

### Participants

Of 1731 allergists contacted, 294 (17.0%) replied to the invitation and 193 of these (11.1% of those contacted) agreed to participate in the study. Ninety of these physicians enrolled at least one patient. All but four physicians considered themselves to have expertise in allergology.

**Fig. 1:**
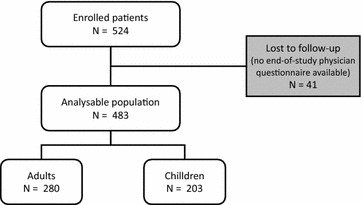
Patient disposition

A total of 524 patients were enrolled into the study, of whom 483 (280 adults and 203 children) were analysable. An end-of-study physician questionnaire was unavailable for the remaining 41 patients (reasons unknown), who did not return for a follow-up consultation and could not be contacted by telephone. These patients thus could not be evaluated and were excluded from the analysis population (Fig. 1). The sociodemographic and clinical characteristics of the analysable population are presented in Table 1. The children were principally boys whilst adults were equally divided between men and women. Allergic rhinitis was associated with conjunctivitis in the majority of cases. Around half of the patients presented other allergic conditions, mostly asthma. During allergic episodes in the previous pollen season, the majority of patients presented persistent symptoms which were moderate to severe according to the ARIA classification [19]. At least one specific grass pollen allergy test had been performed in all patients, a skin prick test in all but four patients and a specific IgE assay in around half. During the previous pollen season, maintenance treatments had been prescribed to three-quarters of the patients and acute treatments to a little under half. In both cases, systemic antihistamines were the most frequently prescribed medication.

**Table 1:** Demographic and clinical characteristics of the analysable population

	Children (N = 203)	Adults (N = 280)	Total (N = 483)
Gender (n,%; male)	129 (63.5%)	139 (49.6%)	268 (55.5%)
Age (mean ± SD; years)	11.8 (3.4)	33.8 (10.9)	24.5 (13.9)
Smoker or ex-smoker (n, %)	3 (1.5%)	74 (26.4%)	77 (15.9%)
Living in an urban environment (n, %)	88 (43.3%)	167 (59.6%)	255 (52.8%)
Age at onset of allergic rhinitis (mean ± SD; years)	7.8 (3.3)	18.6 (9.9)	14.1 (9.5)
Allergic rhinitis with conjunctivitis (n, %)	170 (83.7%)	244 (87.1%)	414 (85.7%)
Other allergic conditions (n, %)			
None	90 (44.3%)	143 (51.1%)	233 (48.2%)
Asthma	84 (41.4%)	96 (34.3%)	180 (37.3%)
Eczema	32 (15.8%)	30 (10.7%)	62 (12.8%)
Food allergy	14 (6.9%)	16 (5.7%)	30 (6.2%)
Urticaria	6 (3.0%)	12 (4.3%)	18 (3.7%)
Other associated chronic disease	9 (4.4%)	20 (7.1%)	29 (6.0%)
Symptom frequency^a^ (ARIA classification) (n, %)			
Intermittent	30 (14.8%)	34 (12.1%)	64 (13.3%)
Persistent	173 (85.2%)	246 (87.9%)	419 (86.7%)
Allergy severity^a^ (ARIA classification) (n, %)			
Mild	15 (7.4%)	4 (1.4%)	19 (3.9%)
Moderate or severe	188 (92.6%)	276 (98.6%)	464 (96.1%)
Specific grass pollen allergy test performed (n, %)	203 (100.0%)	280 (100.0%)	483 (100.0%)
Skin prick test	200 (98.5%)	279 (99.6%)	479 (99.2%)
Anti-grass pollen IgE assay	113 (55.7%)	143 (51.1%)	256 (53.0%)
Nasal irritation test	None	None	None
Maintenance treatment previously prescribed^a^ (n, %)	156 (76.8)	209 (74.6%)	365 (75.6%)
Systemic antihistamines	142 (70.0%)	195 (69.6%)	337 (69.8%)
Topical decongestants and other preparations	62 (30.5%)	85 (30.4%)	147 (30.4%)
Topical corticosteroids	62 (30.5%)	79 (28.2%)	141 (29.2%)
Systemic decongestants and anti-allergic drugs	34 (16.7%)	41 (14.6%)	75 (15.5%)
Inhaled sympathomimetic drugs	21 (10.3%)	12 (4.3%)	33 (6.8%)
Other	13 (6.4%)	14 (5.0%)	27 (5.6%)
Acute treatment previously prescribed^a^ (n, %)	101 (49.8%)	119 (42.5%)	220 (45.5%)
Systemic antihistamines	41 (20.2%)	56 (20.0%)	97 (20.1%)
Topical decongestants and other preparations	39 (19.2%)	37 (13.2%)	76 (15.7%)
Topical corticosteroids	38 (18.7%)	27 (9.6%)	65 (13.5%)
Systemic decongestants and anti-allergic drugs	42 (20.7%)	45 (16.1%)	87 (18.0%)
Inhaled sympathomimetic drugs	26 (12.8%)	21 (7.5% %)	47 (9.7%)

*ARIA* allergic rhinitis and its impact on asthma, *SD* standard deviation

^a^For the previous (2014) pollen season

In the reference sample taken from the EGB database, seventy health insurance beneficiaries were identified who had received a first prescription of 5GPE-SLIT during the study period. Overall, the characteristics of the analysable patients were comparable to those in the EGB sample (Additional file 1: Table S1).

### Treatment by 5GPE-SLIT

**Fig. 2:**
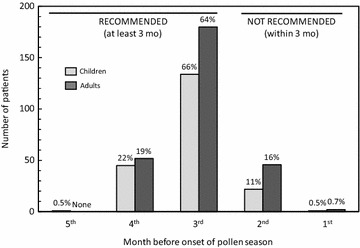
Time between treatment initiation and onset of pollen season

Treatment by 5GPE-SLIT was initiated at least 3 months before the onset of the pollen season, as specified in the prescribing information, in 412 patients (85.3%), principally during the third month (Fig. 2). The mean interval between initiation of 5GPE-SLIT and the onset of the pollen season was 109 ± 16 days.

At least one inconsistency with the recommended prescribing information was found for 13.3% of children and 17.5% of adults, mainly with respect to initiation of 5GPE-SLIT less than 3 months before the start of the pollen season (Table 2).

**Table 2:** Inconsistency of prescription with the prescribing information

	Children (N = 203)	Adults (N = 280)	Total (N = 483)
At least one inconsistency	27 (13.3%)	49 (17.5%)	76 (15.7%)
Children aged < 5 years	None	NA	None
5GPE-SLIT started < 3 mo before pollen season	23 (11.3%)	48 (17.1%)	71 (14.7%)
Symptoms not clinically relevant^a^	4 (14.8%)	1 (2.0%)	5 (6.6%)
No confirmatory diagnostic test of pollen allergy	None	None	None

*NA* not applicable

^a^Intermittent symptoms AND mild severity

The mean duration of treatment by 5GPE-SLIT was 5.6 ± 1.9 months in children and 5.2 ± 2.0 months in adults. Fifty-three patients (11.0%; 18 children and 35 adults) had discontinued 5GPE-SLIT before the beginning of the pollen season, principally within the first 2 months. The principal reason for stopping treatment before the pollen season was the occurrence of an adverse event (Table 3). For the remaining patients continuing treatment, the mean duration of treatment after the onset of the pollen season was 72.9 ± 23.8 days in children and 72.7 ± 24.9 days in adults. At the last follow-up visit, the majority of patients had discontinued 5GPE-SLIT, usually due to the end of the planned treatment course (Table 3). Only two adult patients discontinued treatment during the pollen season due to the occurrence of an adverse event. The proportion of patients still taking 5GPE-SLIT was 64.7% 60 days into the pollen season, 42.5% after 75 days and 19.1% after 90 days.

**Table 3:** Reasons for discontinuation of 5GPE-SLIT before the end-of-study follow-up visit

	Children (N = 203)	Adults (N = 280)	Total (N = 483)
Discontinuation before the start of the pollen season	N = 18	N = 34	N = 52
Planned end of treatment course	1 (5.6%)	2 (5.9%)	3 (5.8%)
Patient decision	2 (11.1%)	3 (8.8%)	5 (9.6%)
Occurrence of an adverse event	14 (77.8%)	27 (79.4%)	41 (78.8%)
Other	1 (5.6%)	2 (5.9%)	3 (5.8%)
Discontinuation after the start of the pollen season	N = 150	N = 191	N = 341
Planned end of treatment course	136 (90.7%)	172 (90.1%)	308 (90.3%)
Patient decision	5 (3.3%)	8 (4.2%)	13 (3.8%)
Lack of efficacy	1 (0.7%)	1 (0.5%)	2 (0.6%)
Occurrence of an adverse event	None	2 (1.0%)	2 (0.6%)
Other	3 (2.0%)	1 (0.5%)	4 (1.2%)
Not documented	5 (3.3%)	7 (3.7%)	12 (3.5%)

Reasons for discontinuation are not mutually exclusive and more than one reason can this be documented in any given patient

### Allergy symptoms during 5GPS-SLIT and comedication

During the 2015 pollen season, 27 children (13.3%) and 64 adults (22.9%) presented persistent allergy symptoms. Allergy severity was rated as moderate-severe for 69 children (34.0%) and 107 adults (38.2%). Around two-thirds of all patients evolved from persistent symptoms or moderate-severe rhinitis in 2014 to intermittent symptoms and mild rhinitis in 2015 whilst taking 5GPE-SLIT (Table 4).

**Table 4:** Evolution of symptom frequency and allergy severity between the 2014 and 2015 pollen seasons

	Children (N = 203)	Adults (N = 280)	Total (N = 483)
Change in symptom frequency between 2014 and 2015			
Not determined in 2015	2 (1.0%)	6 (2.1%)	8 (1.7%)
Improved (persistent in 2014/intermittent in 2015)	146 (71.9%)	181 (64.6%)	327 (67.7%)
Worsened (intermittent in 2014/persistent in 2015)	2 (1.0%)	5 (1.8%)	7 (1.4%)
Unchanged between 2014 and 2015	53 (26.1%)	88 (31.4%)	141 (29.2%)
Change in allergy severity between 2014 and 2015			
Improved (moderate-severe in 2014/mild in 2015)	122 (60.1%)	169 (60.4%)	291 (60.2%)
Worsened (mild in 2014/moderate-severe in 2015)	3 (1.5%)	None	3 (0.6%)
Unchanged between 2014 and 2015	78 (38.4%)	111 (39.6%)	189 (39.1%)

For the most frequent symptoms of rhinitis, around half the patients who had reported individual symptoms during the previous (2014) pollen season, reported that these symptoms were absent during the 2015 pollen season whilst receiving 5GPE-SLIT (Table 5).

**Table 5:** Evolution of symptoms between the previous (2014) and current (2015) pollen seasons

	Children (N = 203)		Adults (N = 283)	
	2014 pollen season	2015 pollen season	2014 pollen season	2015 pollen season
Reported symptoms, n (%)				
Unilateral nose symptoms	27 (13.3%)	23 (11.3%)	35 (12.5%)	53 (18.9%)
Viscous purulent rhinorrhoea	12 (5.9%)	1 (0.5%)	7 (2.5%)	13 (4.6%)
Abundant rhinorrhoea/posterior draining	94 (46.3%)	38 (18.7%)	154 (55.0%)	66 (23.6%)
Facial pain	15 (7.4%)	5 (2.5%)	51 (18.2%)	18 (6.4%)
Recurrent nose bleeds	28 (13.8%)	17 (8.4%)	15 (5.4%)	12 (4.3%)
Hypo-osmia	57 (28.1%)	18 (8.9%)	94 (33.6%)	44 (15.7%)
Reported symptoms most days, n (%)				
Watery rhinorrhoea	191 (94.1%)	102 (50.2%)	276 (98.6%)	130 (46.4%)
Repetitive sneezing	192 (94.6%)	91 (44.8%)	275 (98.2%)	145 (51.8%)
Blocked nose	171 (84.2%)	84 (41.4%)	243 (86.8%)	93 (33.2%)
Nasal pruritus	154 (75.9%)	76 (37.4%)	243 (86.8%)	115 (41.1%)
Signs of conjunctivitis	171 (84.2%)	86 (42.4%)	252 (90.0%)	118 (42.1%)
Symptom frequency, n (%)				
Less than 4 days per week	19 (9.4%)	151 (74.4%)	23 (8.2%)	183 (65.4%)
More than 4 days per week	184 (90.6%)	50 (24.6%)	257 (91.8%)	90 (32.1%)
Symptom duration, n (%)				
Less than 4 weeks	17 (8.4%)	156 (76.8%)	16 (5.7%)	175 (62.5%)
More than 4 weeks	186 (91.6%)	46 (22.7%)	264 (94.3%)	100 (35.7%)
Symptom impact: sleep, n (%)				
Normal	104 (51.2%)	174 (85.7%)	120 (42.9%)	227 (81.1%)
Disturbed	99 (48.8%)	29 (14.3%)	160 (57.1%)	53 (18.9%)
Symptom impact: social and recreational activities, n (%)				
Normal	83 (40.9%)	174 (85.7%)	95 (33.9%)	234 (83.6%)
Disturbed	120 (59.1%)	27 (13.3%)	185 (66.1%)	46 (16.4%)
Symptom impact: professional and/or school activities, n (%)				
Normal	95 (46.8%)	29 (14.3%)	116 (41.4%)	232 (82.9%)
Disturbed	108 (53.2%)	172 (84.7%)	163 (58.2%)	47 (16.8%)
Symptom impact: uncomfortable symptoms, n (%)				
Not particularly uncomfortable	20 (9.9%)	155 (76.4%)	11 (3.9%)	199 (71.1%)
Uncomfortable	183 (90.1%)	48 (23.6%)	269 (96.1%)	81 (28.9%)
Symptom frequency, n (%)				
Intermittent	30 (14.8%)	174 (85.7%)	34 (12.1%)	210 (75.0%)
Persistent	173 (85.2%)	27 (13.3%)	246 (87.9%)	64 (22.9%)
Allergy severity, n (%)				
Mild	15 (7.4%)	134 (66.0%)	4 (1.4%)	173 (61.8%)
Moderate or severe	188 (92.6%)	69 (34.0%)	276 (98.6%)	107 (38.2%)

**Fig. 3:**
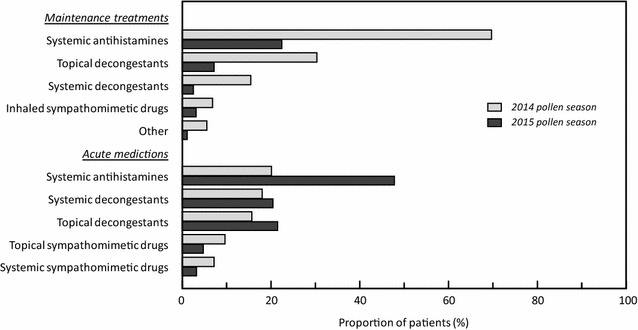
Comparison of prescription rates for acute and maintenance treatments for allergic rhinitis between the 2014 (grey square) and 2015 (black square) pollen seasons

During the 2015 pollen season, 141 patients (29.2%; 53 children and 88 adults) received a maintenance treatment in addition to 5GPE-SLIT. This represents a reduction of 61.4%, in the proportion of patients prescribed maintenance treatments compared to the 2014 season. This reduction was observed for all classes of medication (Fig. 3). This reduction in prescription of maintenance treatments is accompanied by an increase in prescription of acute treatments: 297 patients (61.5%; 126 children and 171 adults) received at least one prescription during 2015, representing an increase of 35.0% compared to 2014. This increase was particularly noticeable for prescription of systemic antihistamines.

The mean treatment impact score was 2.2 ± 1.0 (median 2.3) in children and 2.3 ± 0.9 (median 2.4) in adults. The treatment impact score was ≥ 1, indicating at least some benefits, in 168 children (87.0%) and in 238 adults (90.5%). The individual items of the patient impact score with the largest differences in rating between the inclusion and end-of-study visit were itching in the nose, eyes or palate, relief of runny or stuffed-up nose and relief of all symptoms (Additional file 1: Table S2).

### Tolerability

A total of 255 adverse events were reported by 122 patients during the course of the study. The proportion of patients reporting adverse events was similar in children (23.2%) and adults (26.8%) (Table 6). The majority of these adverse events were considered to be related to 5GPE-SLIT. The most frequent of these related adverse events were throat irritation in 27 patients, oral pruritus in 19 patients and mouth oedema in 12 patients. No other related adverse event was reported in more than ten patients. Eleven events in four patients were classified as serious (two cases of dyspnoea, and one case each of abdominal pain, throat tightness, lip oedema, gingival oedema, swollen tongue, cough, dyspnoea, chest discomfort and oropharyngeal discomfort) and nine events in eight patients were unexpected (not listed in the summary of product characteristics), including two cases of aphthous ulcers and one case each of mouth mucosal rash, gum oedema, papular rash, sensation of suffocating, sensation of foreign bodies in the eye and nasal ulcer. One adverse event (the case of gum oedema) was both serious and unexpected.

**Table 6:** Adverse events reported during the study

Number of patients presenting adverse events (n, %)	Children [N = 203]	Adults [N = 280]	Total [N = 483]
Any adverse event *Number of adverse events*	47 (23.2%) *91*	75 (26.8%) *164*	122 (25.3%) *255*
Adverse events related to 5GPE-SLIT *Number of adverse events*	36 (17.7%) *67*	62 (22.1%) *140*	98 (20.3%) *207*
Unexpected adverse events related to 5GPE-SLIT *Number of adverse events*	3 (1.5%)	5 (1.8%)	8 (1.7%)
Serious adverse events related to 5GPE-SLIT *Number of adverse events*	2 (1.0%)	2 (0.7%)	4 (0.8%) *11*
Serious and unexpected adverse events related to 5GPE-SLIT *Number of adverse events*	None	1 (0.4%)	1 (0.2%)

Italic represents the number of adverse events (one patient could have several dares event, and could be removed for the 3 last items (unexpected adverse events, serious adverse events, unexpected and serious adverse events)

## Discussion

The primary objective of this study was to evaluate the coherence of real-world prescription of 5GPE-SLIT with the prescribing recommendations. We found that this coherence was high, with all conditions of the indication being respected for over eight out of ten patients. There were no breaches of the prescribing information with regard to the lower age limit for prescription to children, nor for the requirement for a positive pollen allergy test. The principal inconsistency with the prescribing information (71 out of 76 cases) related to the initiation of treatment less than 3 months before the onset of the pollen season. However, it should be noted that it is not possible to predict exactly when the pollen season will begin, and this date may vary considerably from year to year depending on meteorological conditions. Prescription of 5GPE-SLIT is restricted in France to physicians trained and experienced in the treatment of allergic disorders. In our sample of 90 physicians, 86 considered themselves to have expertise in allergology.

In terms of effectiveness, we observed that around two-thirds of patients improved from persistent to intermittent symptoms and from moderate-severe to mild disease compared to the previous pollen season. Around half the patients reported the absence of the most frequent symptoms of rhinitis during 5GPE-SLIT treatment. In terms of numbers needed to treat, this would represent a respectable benefit of 5GPE-SLIT. The data are difficult to compare with those obtained from interventional clinical trials, due to the use of different measures of efficacy/effectiveness and much stricter eligibility criteria [21]. However, data from the RNSA pollen surveillance network (http://www.pollens.fr/accueil.php) suggest that the 2015 pollen season lasted somewhat longer than the 2014 season and that similar mean maximum pollen counts were reached in both years. For this reason, it is unlikely that the difference in symptom presentation between the 2 years can be explained by a less intense pollen season in 2015.

Compared to the previous pollen season, the use of other maintenance treatments was reduced by two-thirds. This would be expected since 5GPE-SLIT is prescribed instead of these medications in order to prevent allergic rhinitis symptoms. This reduction is compensated by an increase of prescription of the same medication for acute use on an as-needed basis, notably of systemic antihistamines. This may be a way to ensure that patients have a stock of medication available and ready to use in case of symptom occurrence. In terms of cost-saving, this might be a drawback.

The tolerability of 5GPE-SLIT in this study was acceptable and consistent with what is known of the safety profile of the medication [22]. The most frequent adverse events reported were local oropharyngeal reactions such as throat irritation, oral pruritus and mouth oedema. As expected for an observational study, the rate of adverse event reporting was lower than that reported in interventional clinical trials [22]. Forty-three patients discontinued 5GPE-SLIT due to the occurrence of an adverse event, but this happened before the start of the pollen season for all but two patients. Discontinuation before the start of the pollen season does not deprive the patient of a potentially successful therapy, since an alternative prophylactic treatment can be introduced.

Little published information is available on real-world use of 5GPE-SLIT. In a large multicentre observational study involving over 1400 patients with clinically relevant allergic symptoms caused by grass pollen performed in Germany [23], a fifty percent reduction in a combined rhinoconjunctivitis score compared to the previous pollen season was observed, associated with an improvement in overall health perceptions and a low occurrence of adverse drug reactions. Similarly, in the SMILE study in Spain [24], conducted in 226 patients with allergic rhinoconjunctivitis, a reduction of fifty percent in the proportion of patients with persistent moderate or severe AR was reported following initiation of 5GPE-SLIT, with concomitant improvements in symptoms and quality of life and a reduction in the use of concomitant anti-allergic medications. Our findings are essentially consistent with those of these previous observational studies.

This study has a number of strengths and limitations. The strengths include the relatively large number of patients enrolled, corresponding to around 6% of all patients prescribed 5GPE-SLIT in France during the 2015 pollen season, and the low proportion of patients lost to follow-up (< 10%). In addition, a broad range of clinical variables, and their evolution over the course of the 2015 pollen season, were documented. The principal limitations are those inherent to all observational studies. Given that patients were recruited by a small number of voluntary participating physicians, an inclusion bias cannot be excluded. In this context, it is noteworthy that 68% of patients in the EGB reference sample were prescribed 5GPE-SLIT by their general practitioner, compared to only 24% in our study. The practice of participating physicians may thus not be representative of physicians prescribing 5GPE-SLIT in France. Nonetheless, the characteristics of the patients in our study are similar to those of the patients in the EGB reference sample. A second limitation is that there is no way of ascertaining whether the patients took their 5GPE-SLIT as prescribed, or that other prescription medication evaluated (maintenance and acute treatments) were actually used.

In conclusion, this observational study has shown that, in real-world clinical practice, 5GPE-SLIT is prescribed consistently with the prescribing information and presents a benefit-risk profile to that anticipated from the clinical trial programme.

## Additional file

**Additional file 1.** Additional tables.

## Abbreviations

5GPE-SLIT: five-grass pollen extract sublingual immunotherapy
ARIA: allergic rhinitis and its impact on asthma
CCTIRS: National Advisory Committee on Medical Research Information
CNIL: French national data protection agency
CNOM: National Medical Council
EGB: *Echantillon Généraliste de Bénéficiaires*
HAS: *Haute Autorité de la Santé*
MedRA: Medical Dictionary for Regulatory Activities
NA: not applicable
PBQ: Patient Benefit Questionnaire
PNQ: Patient Needs Questionnaire
RNSA: *Réseau National de Surveillance Aérobiologique*
SCIT: subcutaneous route
SD: standard deviation
SLIT: sublingual route
