# Supplementary material for: An observational cohort study of the use of five-grass-pollen extract sublingual immunotherapy during the 2015 pollen season in France
Source: Allergy Asthma Clin Immunol. 2018 Sep 24;14:38. doi: 10.1186/s13223-018-0262-9 (PMC6151918; doi:10.1186/s13223-018-0262-9)
Supplement: Supplementary file 1 — Additional file 1. Additional tables. [file 13223_2018_262_MOESM1_ESM.docx]

# Additional Material

Additional Table S1. Comparison with the EGB database.

|  | **Analysable population [N = 414]** | **EGB sample [N = 69]** |
| --- | --- | --- |
| Gender (male ; n, %) | 221 (53.4) | 33 (47.8) |
| Age (mean ± SD ; years) | 26.4 ± 13.8 | 28.1 ± 13.7 |
| Medical speciality of prescriber^1^ (n, %) |  |  |
| General practitioner | 101 (24.4) | 47 (68.1) |
| Pneumonologist | 52 (12.6) | 17 (24.6) |
| Allergologist | 337 (81.4) | NA |
| Month when 5GPE-SLIT was initiated |  |  |
| November 2014 | 1 (0.2) | 0 (0.0) |
| December 2014 | 58 (14.0) | 16 (23.2) |
| January 2015 | 304 (73.4) | 28 (40.6) |
| February 2015 | 48 (11.6) | 11 (15.9) |
| March 2015 | 3 (0.7) | 14 (20.3) |
| Duration of treatment by 5GPE-SLIT (days) | ***N = 406*** | ***N = 56*** |
| Mean ± SD | 162.4 ± 53.4 | 173.2 ± 49.4 |
| Median [range] | 176 [1 - 303] | 178.5 [65 - 297] |

Patients in the analysable population are compared with those receiving a first 5GPE-SLIT prescription in the EGB database.

^1^Consultations with paediatricians are not explicitly identified in the EGB database. For this, reason, patients prescribed 5GPE-SLIT by paediatricians are excluded from this comparison.

Additional Table S2. Scores on the Patient Needs Questionnaire-Patient Benefits Questionnaire (PNQ-PBQ).

|  | Children [N = 203] | | Adults [N = 280] | |
| --- | --- | --- | --- | --- |
|  | Needs^1^  Inclusion | Benefits^2^  End of study | Needs^1^  Inclusion | Benefits^2^  End of study |
| To be able to stay outdoors without symptoms | 3.1 ± 0.9 | 2.6 ± 1.2 | 3.4 ± 0.7 | 2.5 ± 1.2 |
| To no longer have a runny or stuffed-up nose | 3.3 ± 0.9 | 2.4 ± 1.3 | 3.5 ± 0.7 | 2.5 ± 1.3 |
| To not have itching on the eyes, nose or palate anymore | 3.3 ± 0.9 | 2.6 ± 1.3 | 3.5 ± 0.8 | 2.5 ± 1.3 |
| To not have burning or watery eyes anymore | 3.2 ± 1.0 | 2.5 ± 1.3 | 3.3 ± 0.9 | 2.6 ± 1.3 |
| To be cured of all symptoms | 3.4 ± 0.7 | 2.7 ± 1.2 | 3.6 ± 0.6 | 2.7 ± 1.2 |
| To be able to breathe through my nose more freely | 3.2 ± 0.9 | 2.6 ± 1.2 | 3.2 ± 0.8 | 2.5 ± 1.2 |
| To feel less fatigued or groggy | 2.6 ± 1.2 | 2.2 ± 1.3 | 2.9 ± 1.1 | 2.3 ± 1.2 |
| To have confidence in the therapy | 3.1 ± 1.0 | 2.6 ± 1.2 | 3.2 ± 0.9 | 2.6 ± 1.2 |
| To have an easily applicable treatment | 3.1 ± 1.1 | 2.9 ± 1.1 | 3.1 ± 0.9 | 2.9 ± 1.1 |
| To be able to sleep better | 2.9 ± 1.1 | 2.3 ± 1.4 | 2.9 ± 1.1 | 2.3 ± 1.3 |
| To experience a greater enjoyment of life | 2.9 ± 1.2 | 2.6 ± 1.3 | 3.1 ± 0.9 | 2.6 ± 1.2 |
| To be able to engage in normal leisure activities | 2.9 ± 1.1 | 2.6 ± 1.3 | 3.0 ± 1.0 | 2.5 ± 1.3 |
| To feel less depressed | 2.2 ± 1.5 | 1.8 ± 1.4 | 2.2 ± 1.3 | 2.0 ± 1.3 |
| To be able to concentrate better at work | 2.8 ± 1.2 | 2.2 ± 1.3 | 2.8 ± 1.1 | 2.4 ± 1.3 |
| To no to have sneezing impulses | 3.1 ± 1.0 | 2.5 ± 1.3 | 3.3 ± 0.8 | 2.5 ± 1.3 |
| To have no fear that the disease will become worse | 2.6 ± 1.3 | 2.6 ± 1.3 | 2.5 ± 1.3 | 2.3 ± 1.4 |
| To be more productive in everyday life | 2.4 ± 1.2 | 2.3 ± 1.3 | 2.7 ± 1.1 | 2.4 ± 1.3 |
| To be less dependent on doctor and clinic visits | 2.2 ± 1.4 | 2.3 ± 1.4 | 2.3 ± 1.3 | 2.3 ± 1.4 |
| To have fewer side effects | 2.6 ± 1.3 | 2.4 ± 1.5 | 2.5 ± 1.2 | 2.3 ± 1.4 |
| To feel less irritated | 2.4 ± 1.3 | 1.9 ± 1.4 | 2.4 ± 1.2 | 2.2 ± 1.3 |
| To have fewer out-of-pocket treatment expenses | 2.4 ± 1.3 | 2.0 ± 1.4 | 2.2 ± 1.3 | 2.0 ± 1.5 |
| To need less time for daily treatment | 2.4 ± 1.3 | 2.5 ± 1.4 | 2.2 ± 1.4 | 2.2 ± 1.4 |
| To feel more comfortable showing yourself | 2.2 ± 1.4 | 2.0 ± 1.5 | 2.3 ± 1.3 | 2.2 ± 1.4 |
| To be less burdened in your partnership | 2.2 ± 1.3 | 2.0 ± 1.5 | 2.4 ± 1.3 | 2.3 ± 1.3 |
| To be able to have a normal sex life | 1.9 ± 1.5 | 1.6 ± 1.4 | 2.2 ± 1.3 | 2.4 ± 1.3 |

**^1^ Needs**: At the start of treatment, the patient is requested to rate each of the 25 treatment items for his individual need on a scale of 0 ‘not important at all’ to 4 ‘very important’.

**^2^ Benefits**: At the end of treatment, the patient is requested to rate the extent beneﬁt has been achieved with the current or at last used treatment for each of the 25 treatment items on a scale of 0 ‘did not help at all’ to 4 ‘helped b=very much’.

Scores are represented as mean values ± SD.
